# VoxelStats: A MATLAB Package for Multi-Modal Voxel-Wise Brain Image Analysis

**DOI:** 10.3389/fninf.2016.00020

**Published:** 2016-06-15

**Authors:** Sulantha Mathotaarachchi, Seqian Wang, Monica Shin, Tharick A. Pascoal, Andrea L. Benedet, Min Su Kang, Thomas Beaudry, Vladimir S. Fonov, Serge Gauthier, Aurélie Labbe, Pedro Rosa-Neto

**Affiliations:** ^1^Translational Neuroimaging Laboratory, Departments of Neurology and Neurosurgery, McGill University Research Centre for Studies in Aging, Douglas Research Institute, McGill UniversityMontreal, QC, Canada; ^2^McConnell Brain Imaging Centre, Montreal Neurological Institute, McGill UniversityMontreal, QC, Canada; ^3^McGill University Research Centre for Studies in Aging, Douglas Research Institute, McGill UniversityMontreal, QC, Canada; ^4^Douglas Hospital Research Center, Douglas Research Institute, McGill UniversityMontreal, QC, Canada; ^5^Department of Psychiatry, McGill UniversityMontreal, QC, Canada; ^6^Department of Neurology and Neurosurgery, McGill UniversityMontreal, QC, Canada; ^7^Department of Epidemiology and Biostatistics, McGill UniversityMontreal, QC, Canada

**Keywords:** voxel-wise analysis, multimodal analysis, longitudinal analysis, generalized linear model, mixed effect model, Alzheimer's disease, ROC analysis

## Abstract

In healthy individuals, behavioral outcomes are highly associated with the variability on brain regional structure or neurochemical phenotypes. Similarly, in the context of neurodegenerative conditions, neuroimaging reveals that cognitive decline is linked to the magnitude of atrophy, neurochemical declines, or concentrations of abnormal protein aggregates across brain regions. However, modeling the effects of multiple regional abnormalities as determinants of cognitive decline at the voxel level remains largely unexplored by multimodal imaging research, given the high computational cost of estimating regression models for every single voxel from various imaging modalities. VoxelStats is a voxel-wise computational framework to overcome these computational limitations and to perform statistical operations on multiple scalar variables and imaging modalities at the voxel level. VoxelStats package has been developed in Matlab^®^ and supports imaging formats such as Nifti-1, ANALYZE, and MINC v2. Prebuilt functions in VoxelStats enable the user to perform voxel-wise general and generalized linear models and mixed effect models with multiple volumetric covariates. Importantly, VoxelStats can recognize scalar values or image volumes as response variables and can accommodate volumetric statistical covariates as well as their interaction effects with other variables. Furthermore, this package includes built-in functionality to perform voxel-wise receiver operating characteristic analysis and paired and unpaired group contrast analysis. Validation of VoxelStats was conducted by comparing the linear regression functionality with existing toolboxes such as glim_image and RMINC. The validation results were identical to existing methods and the additional functionality was demonstrated by generating feature case assessments (t-statistics, odds ratio, and true positive rate maps). In summary, VoxelStats expands the current methods for multimodal imaging analysis by allowing the estimation of advanced regional association metrics at the voxel level.

## Introduction

Research studies based on multiple neuroimaging modalities in the same individual (multimodal acquisition) is becoming increasingly popular due to the widespread availability of imaging techniques such as Magnetic Resonance Imaging (MRI) and Positron Emission Tomography (PET). The availability of ample computational resources permits the widespread use of analytical algorithms performing voxel-wise statistical operations where each voxel is treated as a Region-of-Interest (ROI; Friston, 1995). Several analysis toolkits that support voxel-wise statistical operations have been since introduced, as parts of volumetric image processing software; SPM (Friston, 1995), AFNI (Cox, 1996), FreeSurfer (Fischl, 2012) or as independent toolboxes; BPM (Casanova et al., 2007), multistat (Surfstat; Worsley et al., 2000), VLSM (Bates et al., 2003), RMINC (web: https://wiki.mouseimaging.ca/display/MICePub/RMINC; Accessed 23-11-2015), Neuroimaging in Python (NIPY; Millman and Brett, 2007), glim_image (web: https://github.com/BIC-MNI/glim_image; Accessed 23-11-2015). Nevertheless, in the context of multiparametric analysis, the analytical capacities of these toolboxes are confined by a number of limitations including computational efficiency, the lack or limited support for volumetric statistical covariates, restricted choice of statistical models available, and the inadequate inclusion of sophisticated voxel-wise mathematical operations.

Multiparametric imaging research brings the hope of a comprehensive understanding of the dynamic neurodegenerative processes in the human brain. Imaging has the power to provide longitudinal information regarding the accumulation of toxic proteins in the brain as well as the degeneration associated with disease processes. This information is virtually absent in postmortem evaluations given its intrinsic cross sectional nature. Furthermore, it has been shown that clinical symptoms of neurodegenerative diseases such as Alzheimer's (AD) or Parkinson's disease (PD) constitute a late event on the progression of the disease process given the amount of brain damage present in symptomatic individuals. In the context of longitudinal studies, multiparametric imaging analysis would serve to identify signatures of imminent clinical progression and determine the optimal scenario for a disease modifying intervention. Such information is crucial for designing clinical trials. For instance, the widely accepted pathophysiological model of AD involves a cascade of events initialized by the accumulation of a protein called amyloid (measured by PET ligands such as [^11^C]Pittsburgh Compound B ([^11^C]PIB), [^18^F]Florbetapir) and subsequent neurodegenerative events involving hypo-metabolism [measured by PET ligands such as [^18^F] Fludeoxyglucose ([^18^F]FDG)], atrophy, accumulation of neurofibrillary tangles, neuro-inflammation, and many other neurochemical changes (Jack et al., 2010, 2013) preceding several years before the clinical onset of dementia. Therefore, the aforementioned techniques would have immediate applications not only to model disease progression but also to estimate the efficacy of therapeutic intervention targeting upstream events of the neurodegenerative cascade. Several multimodal imaging studies have evaluated the association between the local amyloid plaque deposition and glucose metabolism using [^18^F]Florbetapir PET and [^18^F]FDG PET images in patients in multiple stages of dementia (Engler et al., 2006; Edison et al., 2007; Cohen et al., 2009; Rabinovici et al., 2010; Furst et al., 2012; Ossenkoppele et al., 2012; Lowe et al., 2014; Altmann et al., 2015; Fletcher et al., 2016). However, these studies were conducted either by focusing on a predefined set of brain regions (Engler et al., 2006; Edison et al., 2007; Rabinovici et al., 2010; Lowe et al., 2014; Altmann et al., 2015) or by using simple voxel wise correlation analysis (Cohen et al., 2009). These studies can benefit by performing their analysis at every voxel incorporating multiple imaging modalities, saving time conceptualizing, defining and extracting values from ROI, and avoiding assumptions regarding specific regions. Furthermore, studies evaluating the interaction between biomarkers (Pascoal et al., 2016) and genetic factors (Benedet et al., 2015) can also take advantage of voxel wise statistical modeling (Furst et al., 2012) with imaging covariates, however; at present, performing voxel-wise statistical analyses and mathematical operations often require utilizing several different specialized toolboxes or modifying the study design to suit the toolboxes available.

In this article we present an approach to multimodal integrative image analyses using VoxelStats statistical framework. This framework facilitates the investigation of neuroimaging data using information from other functional or structural imaging modalities. Furthermore, VoxelStats framework allows probing the interactive and mediating effects between imaging modalities and performing sophisticated mathematical operations at the voxel level. The application of the methods facilitated by VoxelStats provides a powerful tool for studies which require multimodal information including, but not limited to, studies for neurodegenerative disorders as mentioned above, neuropsychiatric disorders, and brain injury.

VoxelStats is a statistical framework for voxel-wise operations written in Matlab with existing support for Nifti-1 (Cox et al., 2004), ANALYZE and MINC v2 (Vincent et al., 2004) volumes. At present, VoxelStats includes several utility functions that can be used in building sophisticated voxel-wise statistical analyses and prebuilt functions to perform voxel-wise general/generalized linear and mixed effects regression with multiple multimodal volumetric covariate support, voxel-wise receiver operating characteristic (ROC) analysis and statistical difference testing. VoxelStats utilizes the Matlab parallel computing toolbox and Matlab distributed computing server to parallelize the operations to increase the efficiency of the analysis. In this article, we describe the overall architecture and the computational steps in the prebuilt functions of VoxelStats, followed by the validation of computational accuracy. Subsequently, we perform a feature case assessment to demonstrate a subset of novel features of VoxelStats.

## Methods and implementation

The primary objective of VoxelStats is to serve as a framework to facilitate image based statistical modeling at a voxel level. Its users can utilize the supplied utility functions to perform trivial tasks such as file input/output, artificial parcellation of data for parallel computation, etc. while the main computational task will be carried out by a built in Matlab function. This architectural design has enabled VoxelStats toolbox to be computationally accurate and highly scalable as a framework to support a vast array of functionality, and would allow for seamless integration into existing Matlab pipelines.

### Steps to increase computational performance

The primary challenges encountered when performing voxel-wise mathematical operations are the computational time and the memory requirement. These challenges have been addressed in existing voxel-wise statistical toolkits by implementing specialized regression algorithms for multi-dimensional analyses. With the architectural design to utilize Matlab's built-in methods to perform the primary operation, VoxelStats has increased the scalability of the framework to support sophisticated statistical operations. However, it has increased the time and memory requirement for the statistical operation.

**Figure 1:**
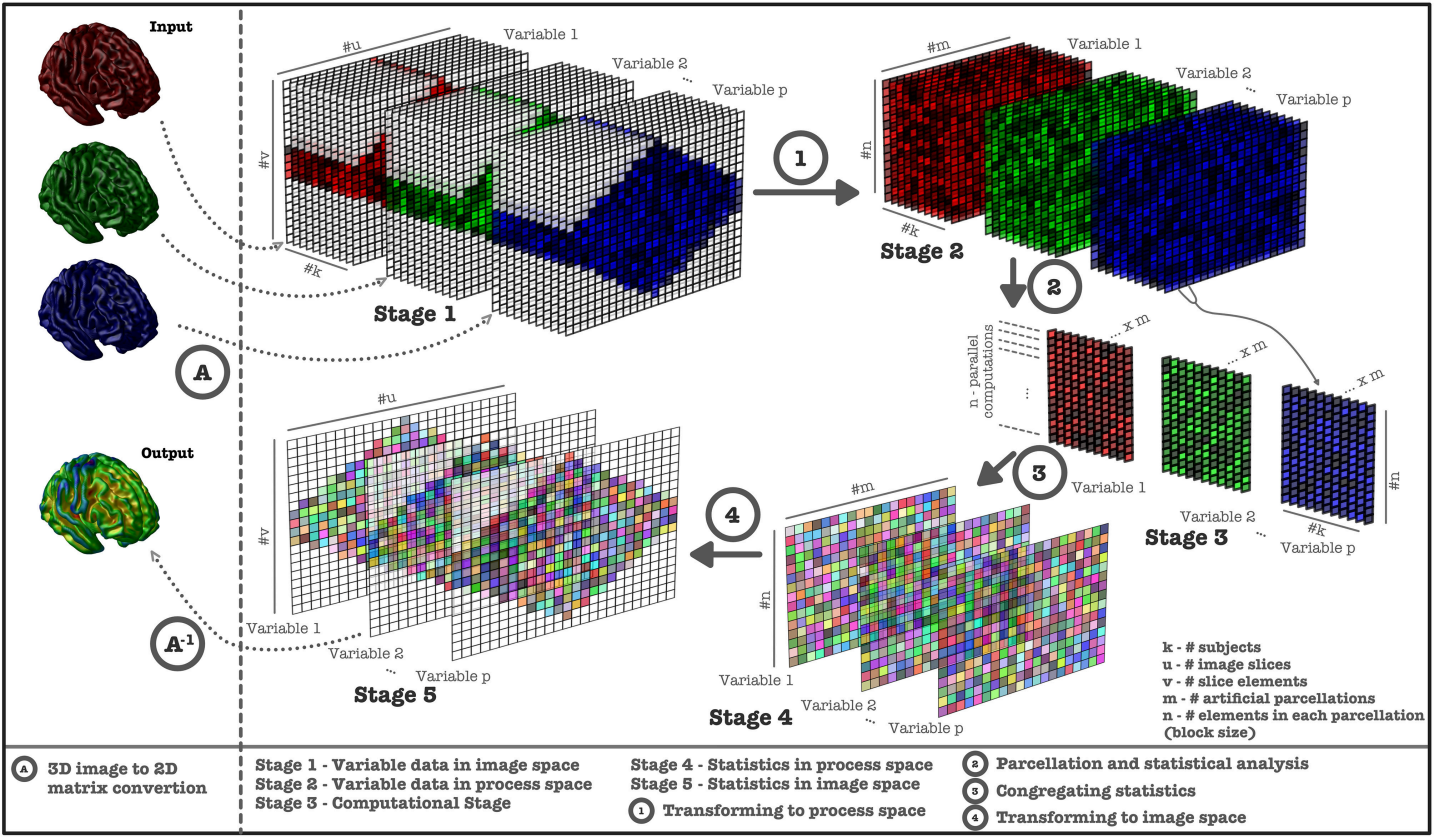
**Computational steps performed in VoxelStats statistical operations**. Image data is retrieved from 3D images and converted (Step A) to a 2D matrix (Stage 1) for each subject. Image data is transformed to process space (Stage 2) using artificial parcellation (Step 1) and statistical operations are performed (Step 2, Stage 3). Subsequently, statistical matrices (Step 3, Stage 4) are generated from the results and transformed back to the 3D image space (Step 4, Stage 5, Step A^−1^).

To overcome the computational time limitation, VoxelStats utilizes data parallelism techniques through the Matlab parallel computing toolbox and the Matlab distributed computing server, with artificial parcellation of data to reduce the memory and network footprint. The artificial parcellation step splits and transforms the masked volumetric data from image space to a process space, which contains a predefined number of parcellations and a uniform number of voxels in each parcellation (Figure 1, see Supplementary Materials for artificial parcellation algorithm).

The number of artificial parcellations has been set to 200 and has been calculated based on the performance of the framework on the development setup; a Matlab computing cluster with 5 nodes/32 workers and volumetric images resampled to the MNI 152 (Fonov et al., 2011) template and operations based on the MNI 152 brain mask. However, depending on the computational setup the user can modify this number to improve the computational performance.

### Procedural steps in prebuilt functions

Prebuilt statistical operations in VoxelStats use the utility functions to perform trivial operations including reading and writing volumetric data and mask information while the computational operations are performed using Matlab's own implementations. Although the manner in which the framework can be utilized is conditional on the analysis performed, the prebuilt functions follow a similar procedural structure differing only to allow function-specific operations.

#### Voxel-wise statistical group difference

Two prebuilt functions are available to perform voxel-wise statistical group differences; one for unpaired *t*-test analysis, which is generally useful in cross-sectional study designs, and the other for paired *t*-test analysis, which is generally used in longitudinal study designs (Hölzel et al., 2011; Soriano-Mas et al., 2011). Although invoking any prebuilt function in VoxelStats requires a specific set of arguments, in most prebuilt functions the user can specify a Matlab string argument to filter a set of samples to be included in the analysis.

Both statistical group difference procedures use this string argument to refine the input samples which will be included in the analysis. This step is followed by the evaluation of the volume information from the provided mask file, which will be used in multiple steps of the analysis including extracting volumetric data from subject files and performing voxel-wise statistical analysis. Subsequently, grouping information is evaluated and the volumetric data is read based on the mask information provided. Unpaired *t*-test function uses Matlab's “ttest2” method to perform the statistical operation while the paired *t*-test function utilizes Matlab's “ttest” method.

#### General/generalized linear and mixed effects regression

Four prebuilt functions are available to perform voxel-wise linear regression analysis, generalized linear regression analysis, and general/generalized mixed effects regression analysis with support for voxel-wise predictor and covariate effects. Analogous to statistical difference procedures, these functions follow the filtering string argument to refine the sample set. This step is followed by parsing the statistical model string to identify the variables used in the mathematical model. Subsequently, the mask information is read, and the volumetric data from the subject files are read based on the variable information and the mask information provided. This step is followed by performing any operation (arithmetic operations such as negation, inverse, scalar addition, or multiplication) specified by the user on the subject data extracted. Subsequently a sample regression (single voxel) is performed to identify the output parameters such as the number of output variables and their names.

Following this step, the extracted voxel data are artificially parcellated, and the voxel-wise statistical operation is performed using Matlab's “fitlm,” “fitlme,” “fitglm,” and “fitglme” functions for linear regression analysis, mixed effects regression analysis, generalized linear regression analysis, and generalized mixed effects regression analysis, respectively, utilizing the Matlab parallel computing toolbox and Matlab distributed computing server.

#### Voxel-wise ROC analysis

The function that performs voxel-wise ROC analysis follows the steps adapted from the regression functions by evaluating the sample filtering string argument mentioned earlier, followed by extracting the mask information and corresponding subject volumetric data. Subsequently, grouping information is evaluated and the extracted volumetric data are artificially parcellated similar to the regression function. Matlab's “perfcurve” method is utilized to carry out the ROC analysis.

### Multiple comparisons correction

Statistical inference based on the results from any massively univariate analysis resulting in voxel wise hypothesis testing must be preceded by multiple comparison correction to reduce the family wise error (FWE). VoxelStats framework provides functionality to perform cluster based multiple comparisons correction based on Random Field Theory (RFT; Worsley et al., 1996), and can be used on results from the prebuilt group difference or regression analysis functions. Using the probability thresholds (*p*-value) provided by the user, VoxelStats calculates the initial cluster threshold (*t*-value) using the T distribution and the cluster size threshold using RFT. These two thresholds are then applied to the statistics images resulting from the analyses. The initial cluster threshold is used to define the clusters by retaining groups of voxels above the threshold. Subsequently, cluster sizes are calculated with a connectivity level of 6, and the cluster size threshold is used to rule out the clusters below the threshold value.

### User interaction

**Figure 2:**
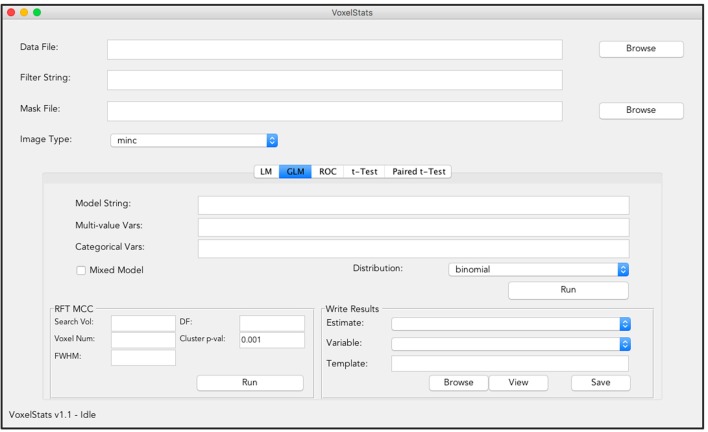
**Graphical User Interface (GUI) of VoxelStats**. Users can use this GUI to perform voxel-wise statistical operations including General/Generalized linear regression, ROC analysis, paired, and unpaired group comparisons. The GUI also includes functionality to preform random field theory based multiple comparison correction and visualization of the results.

**Figure 3:**
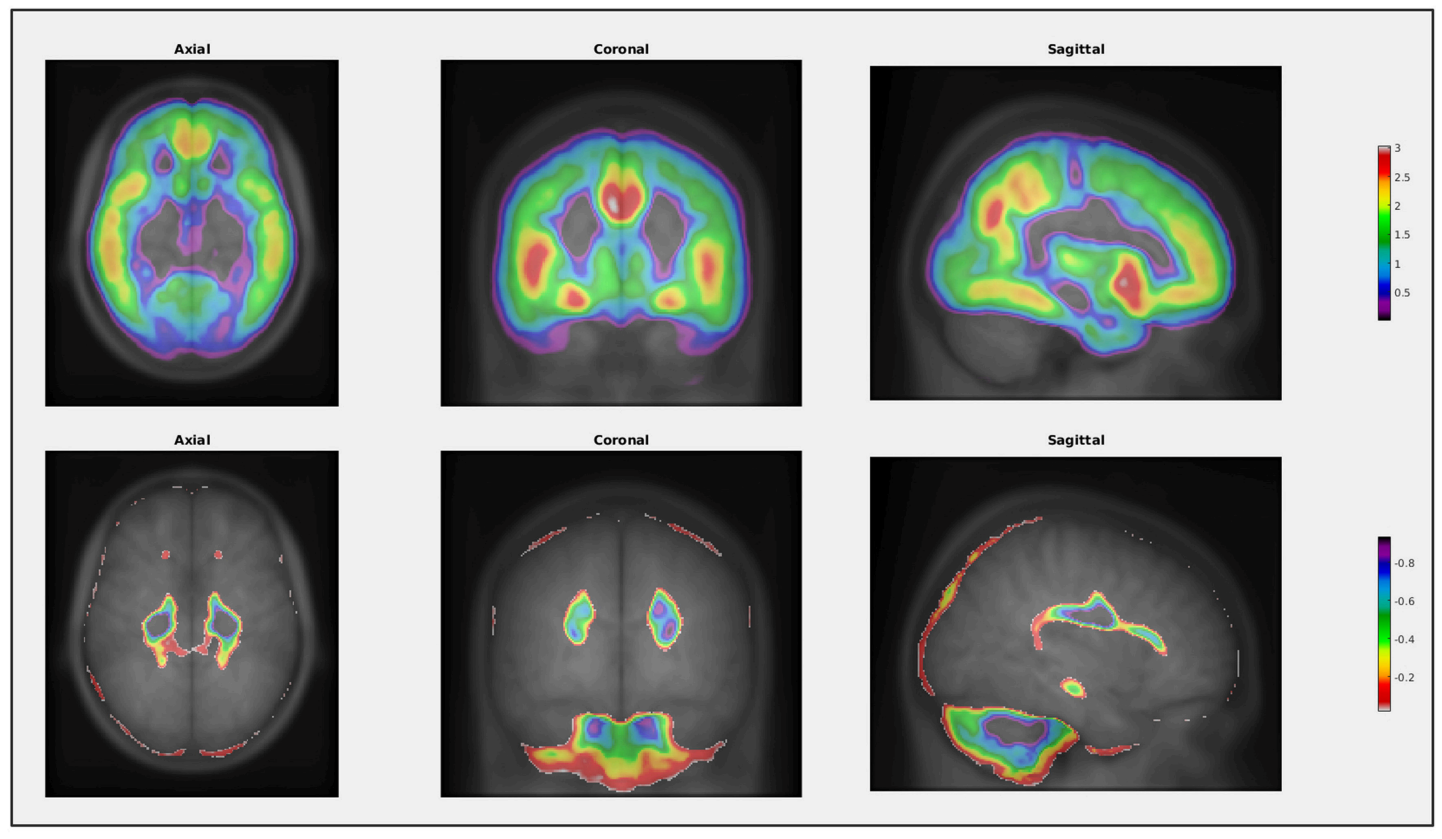
**Example of the visualization function in VoxelStats**. This function can be used to visualize any statistical result from VoxelStats.

Commands to perform statistical operations in VoxelStats are designed to be intuitive and convenient to increase the ease of accessibility and user-friendliness. This reduces the complexity of the functions in VoxelStats, allowing rapid prototyping of statistical hypotheses with minimal programming fluency. In addition, VoxelStats includes a graphical user interface (GUI; Figure 2) to further increase the ease of accessibility. The GUI can be used to perform any prebuilt function mentioned above and to perform multiple comparisons correction on the resultant statistical images. Furthermore, users can visualize these statistical images using the interface provided through the GUI (Figure 3) or the Matlab commands.

### Inputs to voxelstats

At present, inputs to VoxelStats must be 3D volumetric images with the same image resolution (in voxels). This includes any volumetric response variables, predictor variables, covariates, and the image mask. Furthermore, it is expected that all the images are spatially normalized to a common image space and appropriately smoothed. Example input image modalities include, but not limited to, Fractional anisotropy (FA) images, Mean Diffusivity (MD) images, Voxel, or Deformation based morphometric images (DBM or VBM), PET Binding Potential (BP), or Standardized Uptake Value Ratio (SUVR) images, PET, or Single-photon emission computed tomography (SPECT) volume of distribution images and cerebral blood flow images.

### Comparison and additional feature case assessment

To compare VoxelStats with other available toolboxes, neuroimaging data ([^18^F]Florbetapir PET, [^18^F]FDG PET, T1-MRI) were acquired for 273 individuals from the Alzheimer's Disease Neuroimaging Initiative (ADNI) database. Demographic (clinical classification, age, gender) and neurophysiological assessments [mini–mental state examination (MMSE), Clinical Dementia Rating Scale Sum of Boxes scores (CDR-SOB)] were also obtained for the same individuals to be included in the regression models. ADNI was launched in 2003 as a public-private partnership, led by Principal Investigator Michael W. Weiner, MD. The primary goal of ADNI has been to test whether serial MRI, PET, other biological markers, and clinical and neuropsychological assessment can be combined to measure the progression of mild cognitive impairment (MCI) and early Alzheimer's disease (AD).

T1 neuroimaging data were processed using the CIVET image processing pipeline (Zijdenbos et al., 2002; Ad-Dab'bagh et al., 2006) and the Voxel Based Morphometry(VBM) maps were generated using the resulting gray matter density images while the PET neuroimaging data were processed to obtain the SUVR maps with an established image processing pipeline (see Supplementary Materials). All neuroimaging data were then resampled to a grid of 95 × 117 × 99 voxels. Statistical models used for comparison and additional feature case assessments are summarized in Table 1.

**Table 1:** **Summary of the statistical models used to compare and demonstrate the principle feature cases**.

**Usage**	**Statistical model**	**Number of samples**
**COMPARING VOXELSTATS WITH EXISTING TOOLBOXES (VALIDATION STUDY)**		
Linear regression with volumetric dependent variable	$\begin{array}{l} \left[{}^{18}F\right]\text{Florbetapir }{\text{PET}}_{\left[n\times \tau \right]}\sim \beta_{0}\;+\;\beta_{1}\;MMSE_{\left[n\times 1\right]}+\\ \beta_{2}\;Age_{\left[n\times 1\right]}\;+\beta_{3}\;Gender_{\left[n\times 1\right]} \end{array}$	273
**ADDITIONAL FEATURE CASE ASSESSMENT**		
Linear regression with volumetric independent and dependent variables	$\begin{array}{l} {[}^{18}F]\text{FDG }{\text{PET}}_{\left[n\times \tau \right]}\sim \beta_{0}\;+\beta_{1}\;VBM_{\left[n\times \tau \right]}+\\ \beta_{2}\;{\text{Age}}_{\left[n\times 1\right]}\;+\beta_{3}\;Gender_{\left[n\times 1\right]} \end{array}$	219
Logistic regression with volumetric independent variable (binary dependent variable)	$\begin{array}{l} \text{ln}{\left(\frac{\pi }{1-\pi }\right)}_{\left[n\times 1\right]}\sim \beta_{0}\;+\;\beta_{1}\;{[}^{18}F]\text{Florbetapir }{\text{PET}}_{\left[n\times \tau \right]}+\\ \beta_{2}\;Age_{\left[n\times 1\right]}\;+\;\beta_{3}\;Gender_{\left[n\times 1\right]} \end{array}$	273
	*where π* = *Probability*(*clinical progression |X*), *Clinical progression* = *developing dementia in* 24 *months* {1, 0}.	
Linear regression with continuous dependent variable and the interaction two volumetric variables	$\begin{array}{l} \text{CDR}-{\text{SOB}}_{\left[n\times 1\right]}\sim \beta_{0}\;+\;\beta_{1}\left[{}^{18}F\right]\text{Florbetapir }{\text{PET}}_{\left[n\times \tau \right]}+\\ \beta_{2}\;(-1)[18F]\text{FDG }{\text{PET}}_{\left[n\times \tau \right]}\\ +\;\beta_{3}[18F]\text{Florbetapir }{\text{PET}}_{\left[n\times \tau \right]}\text{ x }(-1)[18F]\text{FDG }{\text{PET}}_{\left[n\times \tau \right]}\\ +\;\beta_{4}\;Age_{\left[n\times 1\right]}+\;\beta_{5}Gender_{\left[n\times 1\right]} \end{array}$	219
Voxel-wise ROC analysis	Decision Variable $DecisionVariable=\;\left[{}^{18}F\right]\text{Florbetapir }{\text{PET}}_{\left[n\times \tau \right]}$ Classification Variable = *clinical progression* $True\;Positive\;Rate\;\left(TPR\right)\;=\;\frac{\Sigma \text{True Positive}}{\Sigma \text{Condition Positive}}$ $False\;Positive\;Rate\;\left(FPR\right)=\frac{\Sigma \text{False Positive}}{\Sigma \text{Condition Negative}}$	273

Subscripts indicate the dimensions of each variable in the model. n- number of subjects, τ- number of voxels in the image. The highlighted parameters have been evaluated in the results shown in **Figure 5**.

## Results

Evaluating the computational accuracy of VoxelStats was performed using a linear regression analysis with 273 samples, using volumetric data as the dependent variable. The regression model contained one predictor scalar variable, one continuous scalar covariate and one factor covariate. The result was then compared with other toolboxes available for MINC v2 volumes; glim_image and RMINC. Two parallelization configurations [one to utilize 12 processing cores in a single computational node and the other to utilize 32 processing cores in 5 different computational nodes in a network (see Supplementary Materials)] for VoxelStats have been compared with the aforementioned toolboxes.

**Figure 4:**
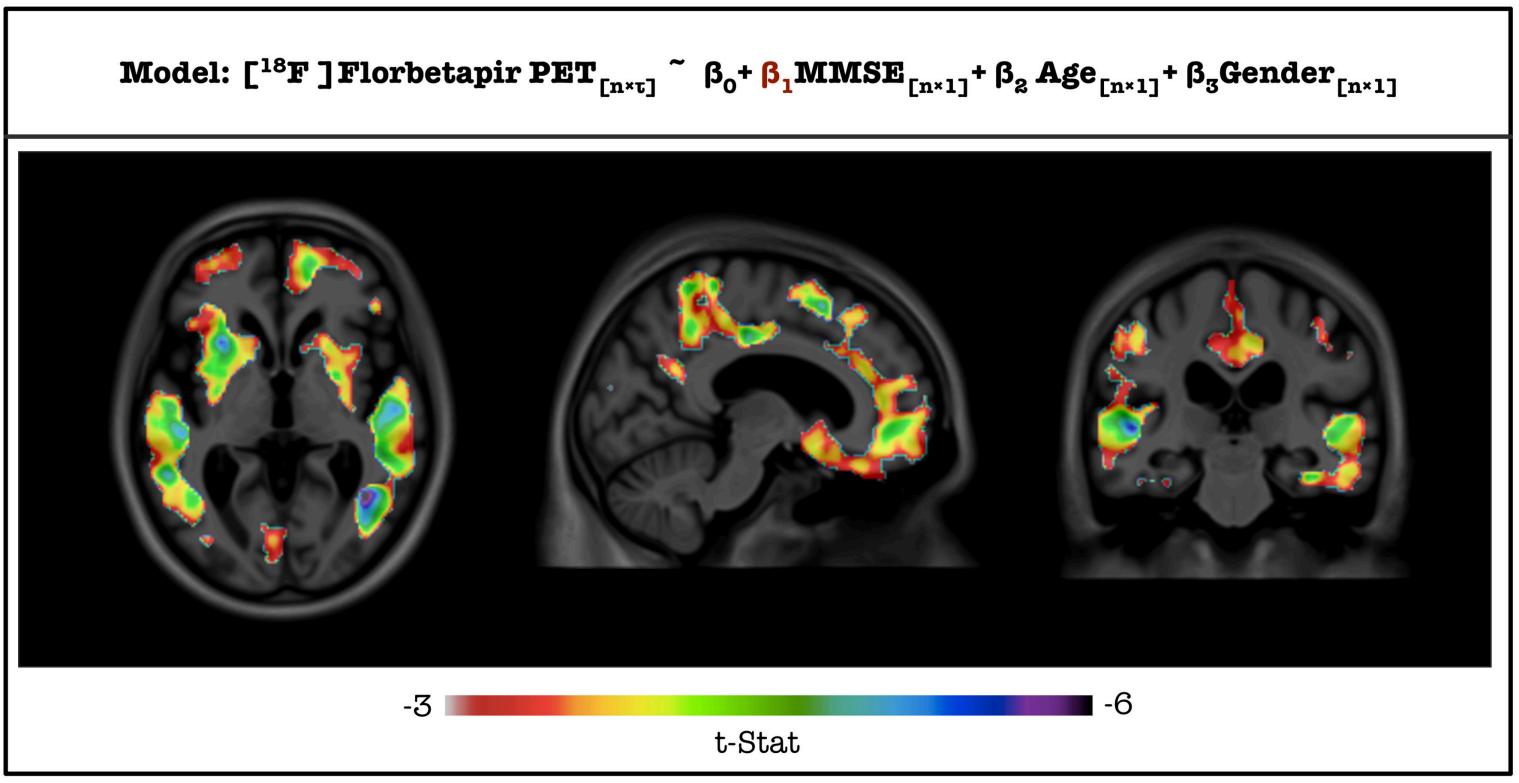
**T-statistical maps for the statistical significance of the parameter for MMSE score generated from VoxelStats toolbox**.

T-statistic image from VoxelStats for the statistical significance of the model parameter (β_1_) for the variable MMSE score is shown in Figure 4. The mean absolute error between the statistical images from VoxelStats and glim_image and VoxelStats and RMINC was 1.730966e-05 and 4.056825e-05, respectively. This indicates no difference between the results obtained from VoxelStats and glim_image or RMINC. Computational times for VoxelStats were 755.8 s and 1655.9 s for 32 processors and 12 processors, respectively (see Supplementary Materials).

**Figure 5:**
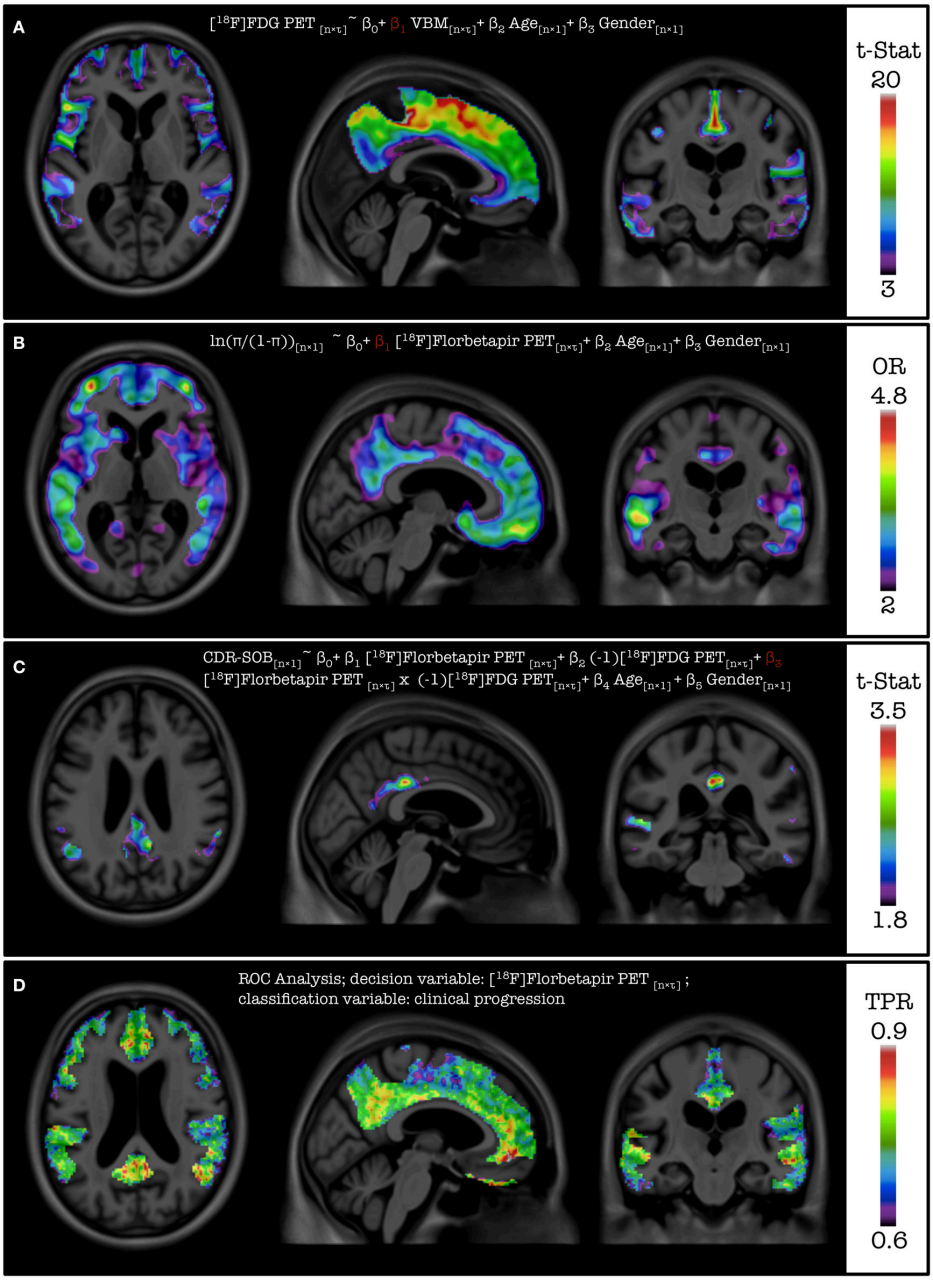
**Results from the feature case assessments**. **(A)** Multiple comparison corrected statistical significance of the parameter for VBM for the association with [^18^F]FDG PET. **(B)** Multiple comparison corrected scaled odds ratio values of developing dementia in 24 months for a unit increase of the standard deviation of [^18^F] Florbetapir PET SUVR. **(C)** Uncorrected statistical significance of the parameter for the interaction between [^18^F]Florbetapir PET and [^18^F] FDG PET for the association with CDR-SOB. **(D)** True positive rate values from the ROC analysis based on [^18^F] Florbetapir PET SUVR in classifying individual developing dementia in 24 months.

Results from the additional feature case analysis of VoxelStats are shown in Figure 5. These include the statistical significance of the model parameter for VBM using linear regression with volumetric independent and dependent variables model, scaled odds ratio map for [^18^F]Florbetapir PET using generalized linear regression with a volumetric independent variable model, statistical significance of the model parameter for the interaction of [^18^F]Florbetapir PET and [^18^F]FDG PET using linear regression with interaction of volumetric variables model, and the true positive rate based on [^18^F]Florbetapir PET for development of dementia using the volumetric ROC analysis. It is important to mention that the parameters of the statistical models are calculated by comparing the same voxel in the dependent and the independent volumetric variables. Although not demonstrated, the utility of the general and generalized regression features can be further expanded with mixed effect modeling to incorporate longitudinal study designs. Statistical maps in Figures 5A,B are corrected for multiple comparisons using the cluster-based correction method. The initial cluster threshold is calculated with *p* < 0.001, and the cluster size threshold is calculated with *p* < 0.05. The statistical map in Figure 5C is not corrected for multiple comparisons.

Figure 5A demonstrates the results of the functionality of inclusion of voxel-wise independent variables on the model by assessing the association between the regional gray matter density measured by VBM and glucose metabolism measured by [^18^F]FDG PET. Based on the images, it can be concluded that the glucose metabolism is associated with the gray-matter density of the particular region. Figure 5C demonstrates the results of the extended functionality of VoxelStats where the association of the interaction of two volumetric measures and a neurophysiological test is assessed. Here the association between the CDR-SOB and the interaction of [^18^F]Florbetapir PET and [^18^F]FDG PET is evaluated, while including Age, Gender, and the main effects of both [^18^F]Florbetapir PET and [^18^F]FDG PET in the statistical model. It should be noted that the [^18^F]FDG PET measures have been negated using VoxelStats variable operations prior to the statistical modeling to increase the interpretability of the interaction analysis, as the expected relationship of the CDR-SOB and [^18^F]FDG PET is inversed. The resultant images highlight that the interaction of [^18^F]Florbetapir PET and [^18^F]FDG PET in brain regions such as the posterior cingulate cortex (PCC) has a positive association with the CDR-SOB test score. Another important feature of VoxelStats is the ability to perform generalized linear modeling with multiple volumetric covariates. This functionality enables the user to perform a vast array of sophisticated association studies using neuroimaging data. Similar to the general linear modeling, these prebuilt functions support any type of dependent variable (voxel-wise or subject wise) and the interaction among any independent variables. Figure 5B demonstrates the results from a logistic regression analysis performed to identify the brain regions which have the highest odds ratios for developing dementia based on [^18^F]Florbetapir PET images. Based on the results, the regions such as the the Precuneus, PCC, parts of the temporal lobe and frontal cortex have the highest odds ratios of developing dementia with the increase of [^18^F]Florbetapir PET SUVR. The ability to perform generalized linear regression enables the user to perform a vast array of association studies using neuroimaging data involving response variables derived from distributions such as Binomial, Poisson, Gamma, or Inverse Gaussian. Another important feature in VoxelStats is the ability to perform voxel-wise ROC analyses to identify the brain regions that have the highest differentiation between two classes based on the classifying performance of an imaging measurement. Figure 5D shows the true positive rate from a voxel-wise ROC analysis performed based on [^18^F]Florbetapir PET images to classify individuals who develop dementia within 24 months. Based on the result, [^18^F]Florbetapir PET measurements from brain regions such as the Precuneus, cingulate, medial frontal, and temporal cortices have the highest classification ability.

## Discussion

VoxelStats is a statistical framework that enables sophisticated voxel-wise operations using multispectral neuroimaging datasets that can be used to answer a multitude of research questions. At present, VoxelStats includes prebuilt functions to perform common statistical operations including general and generalized linear modeling with mixed effects, which can lead to new insights in the analysis of longitudinal neuroimaging data. Its ability to work as an independent Matlab toolbox and the support for Nifti-1, ANALYZE, and MINC v2 format volumes will make VoxelStats immediately useful in the neuroimaging community.

Although ROI-based correction for imaging variables can be considered viable for correcting regional differences, tools similar to VoxelStats will ensure that imaging matrices from one brain region is corrected only for the behavior within the same region. This method also enables voxel-wise independent statistical modeling to assess the relationship between multiple imaging modalities as well as non-imaging measurements such as fluid biomarkers, neurophysiological assessments, and clinical outcomes.

The aforementioned existing toolboxes have limited functionality to perform sophisticated voxel-wise statistical operations, particularly generalized linear modeling and the support for volumetric covariates and their interactions. In Oakes et al. (2007), the authors have modified the multistat algorithm to include a volumetric VBM covariate, the BPM toolbox includes support for general linear regression with volumetric independent variables with volumetric dependent variables, and glim_image can perform general/generalized linear model analysis only with volumetric dependent variables and does not allow imaging covariates. NIPY toolbox currently provides functionality to perform a number of voxel-wise statistical operations including generalized linear modeling, however, its usage is mainly focused toward developers, therefore a neuroimaging analyst might not be able to use it out of the box.

The example used in this article to evaluate the accuracy of VoxelStats is a typical use case in a voxel-wise statistical analysis where a volumetric dependent variable is regressed against one other independent measurement from each individual to identify the brain regions that are associated with the independent measurement. Although the results from VoxelStats are identical to the results from existing toolboxes, it falls behind in time required, due to the architectural design to incorporate built-in Matlab procedures. However, this architectural design has enabled VoxelStats toolbox to be computationally accurate and scalable as a framework to support a vast array of functionalities. The average memory usage of VoxelStats across all the computational nodes during all the analyses was less than 8 GB.

VoxelStats toolbox has many potential uses and while it would not be possible to demonstrate all of the use cases, it is worthwhile to mention the principle feature cases for which VoxelStats can be used (Table 1). Inclusion of voxel-wise covariates in multiple regression is one such feature. The authors of Oakes et al. (2007) and Casanova et al. (2007) have incorporated voxel-wise covariates into a general linear model with a volumetric dependent variable. This analysis design enabled the correction for the regional abnormalities in regression analysis, assessing the independent associations within each voxel. VoxelStats has expanded this functionality by removing the dependency on the volumetric dependent variable and supporting the interaction between any independent variables (voxel-wise and/or subject wise) included in the model (Figures 5A,C). Performing voxel-wise generalized linear regression can be considered one of the prominent features of the VoxelStats framework. This feature allows rapid testing of hypotheses at each voxel, based on imaging measurements where the dependent variable follows a Binomial, Poisson, Gamma, or Inverse Gaussian distribution. The logistic regression example (Figure 5B) in this article is one such example to identify the effect of amyloid deposition in various brain regions for the clinical progression of dementia. The features offered by VoxelStats compared with toolboxes designed to perform voxel-wise linear regression using imaging data is summarized in Table 2. It is important to mention that the information on existing toolboxes was gathered using the relevant publication if available and the publicly available user manuals.

**Table 2:** **Comparison of features offered by VoxelStats with existing statistical software packages designed to perform voxel-wise linear regression using imaging data**.

**Feature in VoxelStats**	**SurfStat**	**BPM**	**glim_image**	**RMINC**
General linear model	✓	✓	✓	✓
Generalized linear models	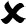	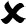	✓	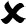
Voxel-wise independent variables	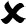	✓	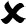	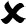 ^a^
Interactions of voxel-wise variables	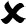	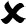	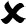	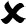
Scalar response variables	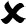	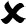	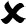	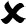
User friendly commands/interface	✓	✓^b^	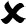	✓
Multiple comparison correction	✓	✓	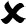	✓
Results visualization	✓	✓	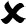	✓
Nifti file format support	✓	✓	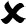	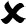
ANALYZE file format support	✓	✓	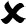	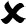
MINC file format support	✓	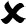	✓	✓

a RMINC currently supports one Voxel-wise independent variable in the regression model, however the model cannot contain any other imaging or scalar covariates.

b Although BPM toolbox provides a user interface, it requires all the imaging and scalar variables and covariates to be listed in separate files.

The voxel-wise ROC analysis based on amyloid-β deposition and the clinical progression can be considered as an example to identify region specific [^18^F]Florbetapir PET thresholds which will be valuable in enrichment of study populations in clinical trials (Carbonell et al., 2015). To the best of our knowledge, VoxelStats is the only analytical package that can perform the voxel-wise ROC analysis, logistic regression with multiple imaging variables and evaluate the interaction between multiple imaging measures at every voxel. It is important to mention that the statistical models performed in this study are merely to demonstrate the functionality of VoxelStats and that the interpretation of the results are beyond the scope of this article.

Due to the amount of statistical computations performed, one of the biggest challenges in VoxelStats is the computational time of the analysis. Although the time required to complete an analysis is acceptable as recorded, it can be further reduced by utilizing the modern grid/cluster computing environments. Other toolboxes developed for neuroimage processing and analysis such as NIAK (web: https://www.nitrc.org/projects/niak/; Accessed 23-11-2015), PSOM (Bellec et al., 2012), and ISC toolbox (Kauppi et al., 2014) have used file based parallelization techniques. These allow the users to utilize cluster computing resources and to overcome the licensing restrictions of Matlab. However, their performance is heavily dependent on the hardware setup of the cluster environment, particularly the speed of data access (Kauppi et al., 2014), and may not be optimum for a task with a large number of disk operations as VoxelStats would require.

One other challenge that needs to be mentioned is the accuracy of the co-registration required between different imaging modalities. As each of the voxel-wise calculation assumes that the information is originated from the same region of the brain, inaccurate or suboptimal image registration will reduce the accuracy of the result. This challenge has been effectively addressed by the advanced image registration algorithms such as DARTEL (Ashburner, 2007), SyN (Avants et al., 2008), IRTK (Schnabel et al., 2001), FLIRT (Jenkinson and Smith, 2001; Jenkinson et al., 2002), AIR (Woods et al., 1992), ANIMAL (Collins et al., 1995), and ART (web: https://www.nitrc.org/projects/art/; Accessed 28-04-2016), which are widely being used for image co-registration. But it is always recommended to perform multiple validation checks to ensure the co-registration accuracy. Interpretability of the result of any general/generalized linear model depends on honoring the primary assumptions: homogeneity, independence, and the normality of residuals. Although performing these tests at a voxel level may not be feasible, the users should evaluate these conditions at least at the level of clusters where significant voxels are present. The users should also consider the problem of multiple comparisons prior to statistical inference and interpretation. A RFT (Worsley et al., 1996) or False Discovery Rate (FDR; Benjamini and Hochberg, 1995) correction method should be used to reduce false positive voxels and to identify the voxels/clusters with statistical significance.

At present, VoxelStats framework supports 3-dimensional volumetric images, therefore analysis using higher dimensional neuroimaging data [functional MRI, dynamic PET, diffusion tensor imaging (DTI)] cannot be performed. However, the support for multidimensional volumetric images are expected to be included in a future release of VoxelStats. VoxelStats also requires the image variables and the image mask used in a single analysis to have the same resolution (in voxels). Users can download the VoxelStats framework as a freely available Matlab library from https://github.com/sulantha2006/VoxelStats/releases/tag/v1.1a1.

VoxelStats framework expands the current multimodal neuroimaging analysis possibilities by enabling the testing of sophisticated image-based hypotheses incorporating multiple imaging modalities simultaneously and response variables from normal, binomial, poisson, gamma, or inverse gaussian distributions. To this extent, VoxelStats framework bests the functional specific or modal specific limitations of existing neuroimaging analysis software.

## Alzheimer's disease neuroimaging initiative

Data used in preparation of this article were obtained from the Alzheimer's Disease Neuroimaging Initiative (ADNI) database (adni.loni.usc.edu). As such, the investigators within the ADNI contributed to the design and implementation of ADNI and/or provided data but did not participate in analysis or writing of this report. A complete listing of ADNI investigators can be found at: http://adni.loni.usc.edu/wp-content/uploads/how_to_apply/ADNI_Acknowledgement_List.pdf.

## Author contributions

SM, TP, SG, and PR were responsible for the tool concept and design. SM and SW were responsible for the implementation of the tool. SM, MS, AB, MK, TB, VF, and PR were responsible for preparing the imaging data used in the article. SM, AL, and PR were responsible for validating the functionality of the tool. SM drafted the manuscript and all authors reviewed and approved the final version of the manuscript.

### Conflict of interest statement

The authors declare that the research was conducted in the absence of any commercial or financial relationships that could be construed as a potential conflict of interest. The handling Editor declared a shared affiliation, though no other collaboration, with one of the reviewers AL and states that the process nevertheless met the standards of a fair and objective review.
