# Supplementary material for: VoxelStats: A MATLAB Package for Multi-Modal Voxel-Wise Brain Image Analysis
Source: Front Neuroinform. 2016 Jun 15;10:20. doi: 10.3389/fninf.2016.00020 (PMC4908129; doi:10.3389/fninf.2016.00020)
Supplement: Supplementary file 1 [file DataSheet1.DOCX]

Supplementary Material

VoxelStats: A MATLAB package for multi-modal voxel-wise brain image analysis.

Sulantha Mathotaarachchi, Seqian Wang, Monica Shin, Tharick Pascoal, Andrea Benedet, Min Su Kang, Thomas Beaudry, Vladimir Fonov, Serge Gauthier, Aurélie Labbe, Pedro Rosa-Neto* for the Alzheimer’s Disease Neuroimaging Initiative.

*** Correspondence:** Corresponding author: Pedro Rosa-Neto, MD, PhD.

Translational Neuroimaging Laboratory, McGill University Research Centre for Studies in Aging,

Douglas Hospital, McGill University, Montreal, QC, Canada.

6875 La Salle Blvd - FBC room 3149, Montreal, QC, Canada H4H 1R3.

Email: pedro.rosa@mcgill.ca

# Supplementary text

Artificial Parcellation

Given: Image matrix $I_{v \times u}$; $u$ number of slices in the image, $v=a \times b$; $a$slice height in voxels, $b$slice width in voxels

Mask matrix $M_{v \times u}$; $u$ number of slices in the image, $v=a \times b$; $a$slice height in voxels, $b$slice width in voxels

Number of artificial parcellations, $m$

1. Total models $\tau= \sum_{u} \sum_{v} M_{uv}$

2. Max models in parcellation $n_{max}= \left\lceil\tau/m \right\rceil$

3. Image Vector $V_{1 \times\tau}= \left\{ I_{\alpha\beta} \right\} \forall\alpha\in\left\{ 1, \ldots, u \right\}; \beta\in\left\{ 1, \ldots, v \right\}; M_{\alpha\beta}=1$

4. Do for $c=1\ldots m$

Index $i_{c}= c \times n_{max}$

If $(i_{c-1}+1$> $\tau)$ Then

Parcellation $P_{c}$, 0

ElseIf $(\left( i_{c}> \tau\right) \& \left( i_{c-1}+1< \tau\right))$ Then

Parcellation $P_{c}$, $\{V_{i_{c-1}+1}, \ldots,V_{\tau}\}$

Else

Parcellation $P_{c}$, $\{V_{i_{c-1}+1}, \ldots,V_{i_{c}}\}$

End If

Each iteration of all the parcellations are followed by the voxel wise computation which is performed in parallel using Matlab’s ‘parfor’ procedure.

# Supplementary Figures and Tables

## Supplementary Figures


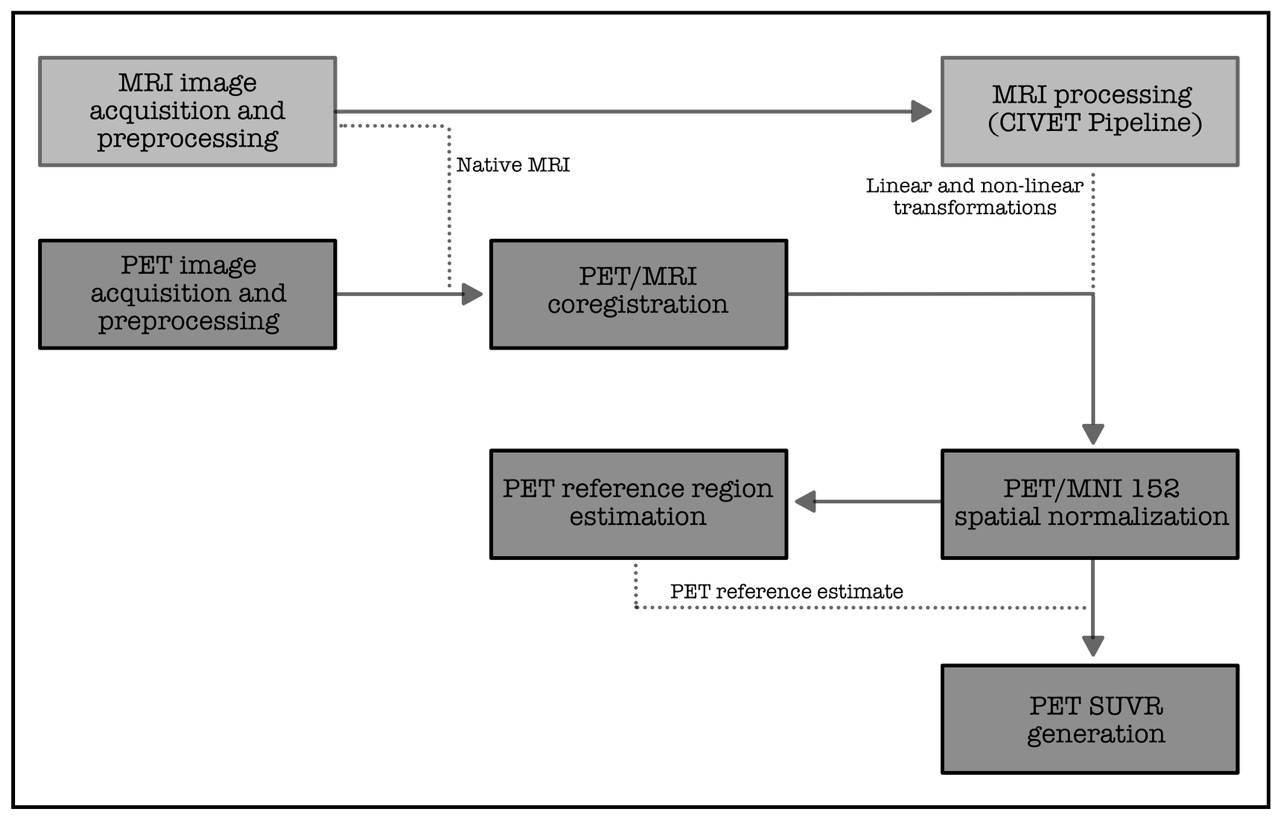


**Supplementary Figure 1.** PET image processing steps used to process [^18^F]Florbetapir PET and [^18^F]FDG PET images. Reference region used for [^18^F]Florbetapir PET and [^18^F]FDG PET are cerebellum grey matter and pons respectively.


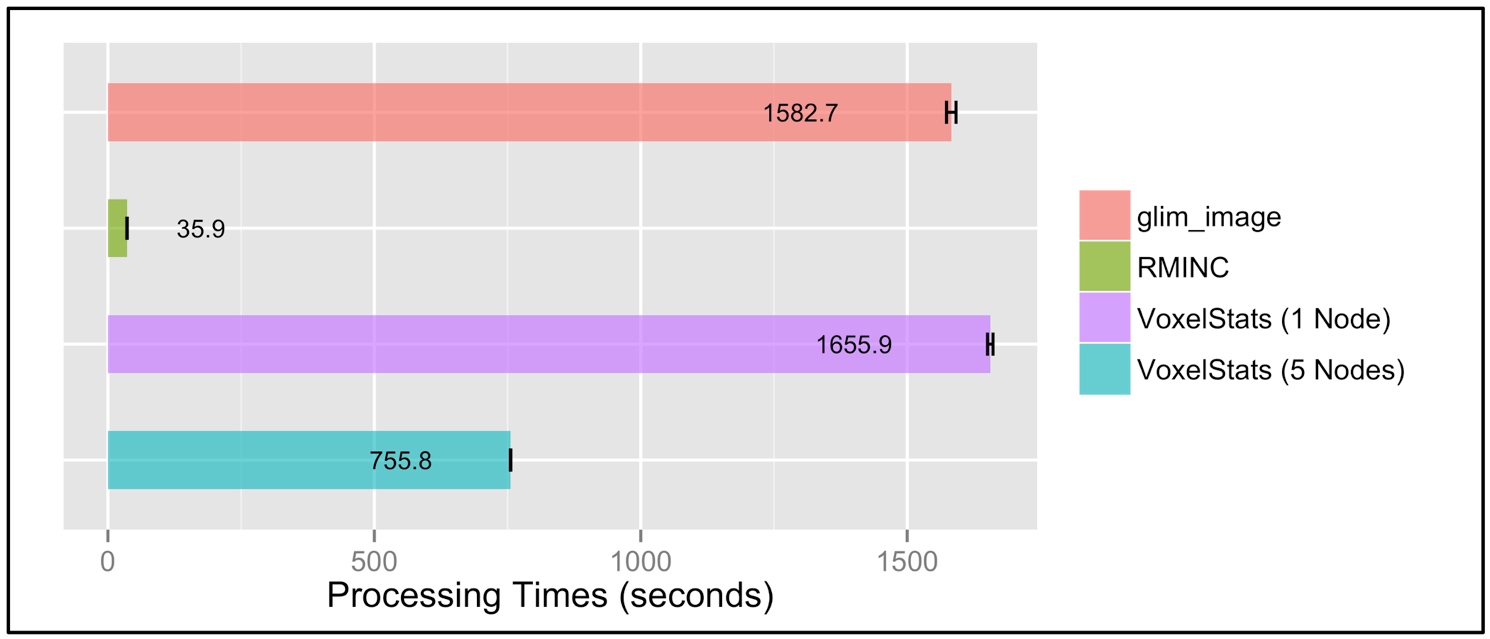


**Supplementary Figure 2:** Processing times of glim_image, RMINC and VoxelStats to complete the statistical model used for comparison. Times have been calculated based on 10 simulations.

## Supplementary Tables

**Supplementary Table 1.** Examples for the commands used in VoxelStats to perform voxel-wise statistical operations.

| Operation | Command |
| --- | --- |
| Linear regression | VoxelStatsLM*(<file type>, <model string>, <csv file>, <mask file>, <voxel variables>, <factor variables>, <subset string>*); |
| Logistic regression | VoxelStatsGLM*(<file type>, <model string>, <distribution>, <csv file>, <mask file>, <voxel variables>, <factor variables>, <subset string>*); |
| Voxel-wise ROC analysis | VoxelStatsROC*(<file type>, <csv file>, <measurement variable>, <class variable>, <mask file>, <subset string>*); |
| *<csv file> - Comma separated file with data*  *<mask file> - Mask file with region to perform analysis*  *<file type> - ‘minc’ \| ‘nifti’*  *<model string> - Eg: ‘Conversion ~ FDG_PET + Age + Gender’*  *<distribution> - Distribution of the response variable (‘binomial’\| ’normal’\| ’poisson’\| ’gamma’\| ’inverse gaussian’)*  *<subset string> - String argument to subset samples from CSV file* | |

**Supplementary Table 2.** Summary of the node specifications used in testing

| Node type | Master Node (N = 1) | Worker Node (N = 5) |
| --- | --- | --- |
| Analysis run | glim_image  RMINC  VoxelStats (1 Node)  VoxelStats (Cluster) – master node. | VoxelStats (5 Nodes) – worker nodes |
| Specification | CPU: 2x Intel® Xeon® X5650 @ 2.67GHz  RAM: 48 GB  Processing Cores: 12 (2x 6)  Virtual Cores: 24 (2x 12)  OS: Ubuntu 14.04 LTS  Matlab Version: 2015a | CPU: 2x Intel® Xeon® E5-2630 @ 2.3GHz  RAM: 64 GB  Processing Cores: 12 (2x 6)  Virtual Cores: 24 (2x 12)  OS: Ubuntu 14.04 LTS  Matlab Version: 2015a |
